# The Evolving Role of Artificial Intelligence in Pediatric Asthma Management: Opportunities and Challenges for Modern Healthcare

**DOI:** 10.3390/jpm16010043

**Published:** 2026-01-08

**Authors:** Valentina Fainardi, Carlo Caffarelli, Susanna Esposito

**Affiliations:** Paediatric Clinic, Department of Medicine and Surgery, University of Parma, 43123 Parma, Italy

**Keywords:** asthma, artificial intelligence, machine learning, precision medicine, digital health

## Abstract

Asthma is a common chronic disease in children, contributing to significant morbidity and healthcare utilization worldwide. The integration of artificial intelligence (AI) and machine learning (ML) into pediatric asthma care is rapidly advancing, offering new opportunities for early diagnosis, risk stratification, and personalized management. AI-driven tools can analyze complex clinical, genetic, and environmental data to identify asthma phenotypes and endotypes, predict exacerbations, and support timely interventions. In pediatric populations, these technologies enable non-invasive diagnostic approaches, remote monitoring through wearable devices, and improved medication adherence via smart inhalers and digital health platforms. Despite these advances, challenges remain, including the need for pediatric-specific datasets, transparency in AI decision-making, and careful attention to data privacy and equity. The integration of AI in pediatric asthma care and into the clinical decision system can offer personalized treatment plans, reducing the burden of the disease both for patients and health professionals. This is a narrative review on the applications of AI and ML in pediatric asthma care.

## 1. Introduction

### 1.1. Asthma as a Global Health Concern

Asthma represents a significant challenge to public health worldwide, impacting people of all ages and backgrounds. According to the Global Asthma Report, the prevalence of asthma stands at 9.1% among children, 11.0% among adolescents, and 6.6% among adults [1]. The prevalence of asthma varies widely between countries and regions. Such disparities are largely influenced by genetic factors, environmental exposures, and differences in healthcare access, but the prevalence of asthma has continued to rise steadily all over the world. Asthma-related morbidity and mortality contribute substantially to global public health burdens. When asthma is inadequately managed, individuals may experience frequent hospital admissions, increased absenteeism from school or work, and a diminished quality of life [2].

Therefore, optimising asthma management and control is vital to mitigating its overall impact. Effective strategies include early and right diagnosis, identifying those at increased risk of exacerbation, improvement of adherence to treatment, managing timely exacerbations, monitoring patients, and selecting appropriate and personalized treatment plans [3]. Artificial intelligence (AI)-driven tools have the potential of implementing these strategies.

### 1.2. The Transformative Role of Artificial Intelligence

In recent years, the role of AI in medicine has been rapidly expanding, revolutionizing various aspects of healthcare and improving the efficiency and accuracy of diagnosis and treatment.

At present, AI technology is part of diagnostic procedures, helping identify diseases more quickly and accurately using advanced imaging and pattern recognition. For example, computer reading of electrocardiograms or analysis of cutaneous lesions has become a reality and been integrated into daily practice. AI is also used to retrieve a huge amount of medical information to diagnose rare and common diseases.

These processes often require a person who supervises the interpretation of these data, but by learning, reasoning, and self-correcting, AI can provide valuable insights to healthcare professionals. AI applied to machine learning (ML) shows promise in many different aspects of healthcare. ML is either supervised, using algorithms based on existing categories, or unsupervised, where algorithms create new patterns or categories independently [4].

ML algorithms help identify at-risk patients, improve the accuracy of diagnosis in complex medical conditions, make predictions, and develop personalized treatments by analysing vast amounts of genetic, clinical, and environmental data. By processing large healthcare datasets, including the omics, ML reveals patterns that might be missed by humans [5,6,7].

Within this context, AI offers profound opportunities to reshape asthma care. The integration of AI into asthma management means the advent of precision medicine, where complex datasets, including clinical, genetic, and environmental information, will be leveraged to deepen understanding of asthma’s heterogeneity and progression. AI can analyze these “big data”, uncovering different pheno- and endotypes, departing from the “one-size-fits-all” approach and moving towards tailored-made precision medicine with the potential of offering personalized treatments to different patients.

AI-driven technologies may not only enhance diagnostic accuracy by identifying underlying patterns but can also predict acute exacerbations and improve asthma control. The prevention of asthma attacks can be achieved through specific AI-based models that integrate data coming from apps and wearable technology like lung function, oxygen saturation or respiratory rate. In addition, these systems can promote communication between patient and physician, leading to earlier interventions and targeted therapy.

The digital evolution of AI in asthma also translates into active support of the patient in performing therapies, therefore improving asthma control. The use of smart inhalers promotes the correct use of the devices and a good inhalation technique. This can reduce the individual burden associated with asthma and supports the development of proactive, patient-centered strategies that adapt to the dynamic nature of this condition. Herein, we will review the applications of AI in asthma care.

### 1.3. Methods

In this narrative review of the literature, we analyzed the current evidence in the field of AI and ML in pediatric asthma. Searches were performed in PubMed up to September 2025. Search terms included AI and ML in combination with asthma and children. Case reports, case series, original research studies, review articles, letters to the editor, randomized controlled trials (RCTs), non-RCTs, and cohort studies (prospective or retrospective) were considered. Only studies reporting on subjects aged 0–18 years were considered, and the search was limited to results in English.

## 2. AI Applications in Asthma Management

### 2.1. Diagnosis and Pheno-Endotyping of Asthma

Starting from the diagnosis, AI integrated into digital stethoscopes can help the clinician to recognise asthma, especially in young children, in low-resource settings, or where long distances prevent rapid in-person clinical examinations [8]. A systematic review found that AI algorithms integrated into stethoscopes can accurately diagnose pediatric respiratory diseases from cough sounds, often outperforming traditional methods [9].

Asthma is a heterogeneous disease with overlapping and evolving phenotypes [10]. Traditional phenotypes are usually described on the basis of parental reports and clinical features, and these do not always reflect the underlying biological mechanism (the endotypes), leading to incorrect treatments. Identifying endotypes and therefore the molecular pathogenesis of asthma can be very helpful in understanding the disease and guiding treatment, but especially in children, this can be challenging [10,11]. In children, induced sputum is difficult to obtain, and bronchoscopies are often procedures reserved for tertiary specialized centers. Non-invasive biomarkers are needed to better understand the underlying mechanism of asthma pathogenesis and to assess response to treatment.

AI with ML can analyze several factors like medical history, exacerbations, biomarkers, clinical signs, pulmonary function, comorbidities, genomics, allergic status, and environmental conditions in an unsupervised manner to accurately describe different clusters of patients. These large, multi-dimensional datasets, including both clinical and molecular features, can uncover hidden patterns that traditional methods cannot detect. 

The repeated analysis of these data can also predict the evolution of the disease and build predictive models for proactive and personalized asthma treatment. AI and ML challenge the traditional ‘one-size-fits-all’ method, where all patients are treated in the same way and can enable tailored treatments based on individual data.

Data-driven techniques, including a certain number of variables, can uncover endotypes using statistical and machine learning techniques such as latent class analysis (LCA) [12] or cluster analysis [13].

In children with severe wheeze where airway inflammation cannot distinguish between episodic viral and multiple-trigger wheeze, the data-driven unbiased analysis of bronchoalveolar lavage revealed four different clusters described as (1) atopic (17.9%), (2) nonatopic with a low infection rate and high use of inhaled corticosteroids (31.3%), (3) nonatopic with a high infection rate (23.1%), and (4) nonatopic with a low infection rate and no use of inhaled corticosteroids (27.6%) [14]. The identification of four clusters suggested that each cluster may respond to different treatments. The combination of objective biomarkers like sensitization, peripheral eosinophilia, and lower airway microbiology may be used as potential biomarkers to stratify treatment.

Since clustering patients with clinical features alone cannot give information on the underlying biologic mechanism, the Unbiased Biomarkers for the Prediction of Respiratory Disease Outcomes (U-BIOPRED) study performed with transcriptomics and genomics an unsupervised clustering of differentially expressed genes in the sputum of 104 eosinophilic and non-eosinophilic adult asthma patients to identify the driving mechanisms that lead to the different inflammatory profiles. Unbiased hierarchical clustering of 508 differentially expressed genes identified three distinct transcriptome-associated clusters (TACs). TAC1 was distinguished by a predominant Th2 signature, including elevated expression of interleukin (IL)-33, innate lymphoid cells (ILC) type 2, and IL-13 genes, and was associated with eosinophilic airway inflammation and severe asthma. TAC2 was characterized by increased expression of interferon and tumour necrosis factor-α genes, along with neutrophilic airway inflammation. TAC3 displayed enrichment for genes involved in metabolic pathways and mitochondrial function, exhibited moderately elevated sputum eosinophils, and maintained better preserved lung function [15].

A transcriptomic analysis of sputum samples after 1 year showed that severe asthma molecular phenotypes were unstable in about half of patients, with many shifting from TAC1 or TAC3 to TAC2 [11]. Hence, underlying mechanisms of asthma can vary in certain subgroups of patients, suggesting that treatment may be less effective over time and needs to be changed accordingly.

Identifying these molecular profiles will help characterize patients and guide the selection of the most effective, personalized treatment plan.

However, the derived clusters may not always describe reliable asthma endotypes. The different methods used in ML algorithms or the use of different algorithms for the same database can modify the results [16]. Furthermore, analyzing the data in a supervised or unsupervised manner can also affect the findings. In children, wherein studies often include small cohorts of patients, heterogeneity can also influence the differences between different studies.

### 2.2. Identification of Subjects at Risk of Asthma Development

Each individual follows their own lung function trajectory starting in childhood, going through to adulthood and old age. This trajectory can be influenced by factors like smoke exposure or effective disease management in patients with asthma. Those with the lowest lung function trajectories are at higher risk of developing chronic obstructive pulmonary disease (COPD) in later life [17].

Identifying individuals at risk of asthma may enable timely intervention during the so-called “window of opportunity,” a period during childhood in which lung function trajectory may still be influenced and eventually improved. Accurately predicting asthma onset prior to clinical manifestation presents a significant opportunity for effective primary prevention of the disease. ML algorithms can predict childhood asthma at 5 years by analyzing early-life risk factors collected from birth cohort studies; the method can identify these factors as targets for intervention. Using the CHILD Cohort Study, 132 variables were measured in 1754 children followed up since birth to 4 years of age. Wheezing, atopy, antibiotic exposure, lower respiratory tract infections, and maternal asthma were the strongest predictive factors for asthma diagnosis at 5 years [18].

Hu et al. assessed a predictive real-life model for early detection of asthma in preschool-aged children by employing seven distinct ML algorithms that incorporated impulse oscillometry, fractional exhaled nitric oxide (FeNO), and various clinical characteristics. Six asthma predictors were identified: wheezing frequency, allergy history, FeNO, airway resistance (R5), and airway reactance (X5) at 5 Hz. The authors then provided the clinicians with an online tool including all of these traits to predict asthma risk in young patients who present with wheezing [19].

A recent meta-analysis of 89 studies examining childhood asthma and allergy trajectories through ML techniques identified four primary patterns: early-onset persistent, mid-onset persistent, early-onset early-resolving, and early-onset mid-resolving wheezing. Male sex was linked to an increased risk of most wheezing trajectories; both prenatal and postnatal exposure to tobacco smoke were similarly associated with all of the trajectories, highlighting the massive negative impact of smoke exposure on wheeze development. Additionally, lower respiratory tract infections during infancy were correlated with most wheezing trajectories [20].

Liu et al. conducted a study evaluating an ML model’s capability to predict future chronic lung diseases, including asthma and COPD, using gut microbiome data from adult subjects. The results demonstrated that the model achieved greater predictive accuracy compared to models relying solely on conventional risk factors [21]. Multiple sources of data can better identify at-risk subjects and potentially suggest primary preventive treatment.

Variables used by AI algorithms to describe asthma patients are listed in Table 1.

### 2.3. Assessment of Exacerbation Risk

AI facilitates the analysis and interpretation of extensive datasets, thereby supporting clinicians in identifying patients at higher risk, recommending tailored treatments, and tracking therapeutic responses in real time. Devices equipped with AI can provide ongoing outcome prediction and further enable the customization of treatment strategies.

The variability in asthmatic patients makes this condition particularly suitable for predictive and targeted therapeutic approaches.

Numerous studies have established the efficacy of ML in forecasting asthma exacerbations. Gorham and colleagues conducted a retrospective analysis involving 26,008 pediatric patients with asthma aged 2 to 18 years, resulting in the development of the Asthma Emergency Risk (AER) score, an algorithm designed to predict emergency department visits due to asthma attacks.

The AER score was determined based on eight distinct predictors: (1) use of inhaled steroids, (2) age under 5 years, (3) asthma-related emergency department visits within the previous year, (4) diagnosis of moderate-to-severe asthma, (5) oral steroid use in the past year, (6) non-asthma-related emergency department visits in the past year, (7) persistent asthma, and (8) asthma-related primary care visits within the last year [23].

Association rule mining, an interpretable method for analysing the effects of multiple exposures, was employed to develop an algorithm designed to predict the risk of asthma attacks in relation to outdoor air quality. The authors determined that exposure to ozone, and particularly to chemical mixtures on the day preceding an asthma attack, was associated with increased harm and a higher likelihood of asthma exacerbations [27]. These findings confirm that exposure to ozone and other chemicals is harmful for young asthmatic subjects. The use of apps with an alert system can be very helpful to monitor air quality and advise when the level of pollution is harmful for asthmatic subjects.

Hurst et al. investigated the effectiveness of a model to predict asthma exacerbations in 5982 pediatric asthma patients. This model integrates spatiotemporally detailed climate data, such as pollution, allergens, and influenza case numbers, with common individual electronic health records (EHRs). The strongest predictive factor identified was having had an asthma exacerbation in the previous year [28]. These results carry significant implications for the development and deployment of clinical decision support systems to identify patients who may benefit from targeted interventions to prevent asthma exacerbations.

A study involving 60,302 adults with asthma used an ML algorithm trained on outpatient data (demographics, comorbidities, laboratory results, and medications) to predict non-severe asthma exacerbations that would require a course of oral glucocorticoids, emergency department visits, and hospitalizations. Risk factors associated with all three outcomes included younger age, the use of long-acting β agonists, high-dose inhaled steroids, and a prior course of oral steroid therapy. Subgroup analysis of 9448 patients with spirometry data identified low forced expiratory volume in the first second (FEV_1_) and a reduced FEV_1_/forced vital capacity (FVC) ratio as prominent risk factors for asthma exacerbation, emergency department visits, and hospitalization [24]. Bose et al. developed several ML models to distinguish between children with early-life asthma diagnoses who would continue to experience persistent asthma symptoms beyond 5 years of age and those who would not. The data for this study were obtained from the EHR of 9934 pediatric patients. Given clinical information up to age 5 years, five ML models were trained to differentiate individuals without further asthma-related visits (i.e., transient diagnosis) from those with asthma-related visits between ages 5 and 10 (i.e., persistent diagnosis). Important identified features were age of last asthma diagnosis under 5 years, total number of asthma-related visits, self-identified Black ethnicity, allergic rhinitis, and eczema. The authors concluded that ML models demonstrate strong predictive capabilities in identifying individuals at risk of persistent asthma. These models could potentially assist clinicians and parents in making informed decisions regarding early childhood asthma [22].

A recent meta-analysis systematically assessed and quantified the performance of ML algorithms in predicting the risk of hospitalization and emergency department admission for acute asthma exacerbations in children. The authors concluded that ML models can accurately predict asthma exacerbations and therefore be extremely helpful in helping parents and clinicians in predicting and treating these events. However, future studies are needed to improve their generalizability through external validation, as well as their successful applicability in clinical practice [29].

A study utilizing wearable technology in children with asthma demonstrated that home monitoring of physiological parameters aligned closely with pediatrician-assessed asthma control. The resulting multivariate model was able to identify nearly 90% of all cases of uncontrolled asthma. Statistically significant differences were observed between controlled and uncontrolled asthma in terms of lung function variability, wake-up time, reliever medication usage, and respiratory rate recovery time following exercise [26].

Although cost and access to wearable devices may currently restrict the applicability of such studies in real life, the rapid integration of technology in our daily lives could soon make them widely available.

A recent pilot study involving young children with recurrent wheezing used both supervised ML and decision tree models to predict future wheeze attacks. The study found that an eosinophil value greater than 4% obtained through finger-prick sampling, along with a symptom score below 75 on the Test for Respiratory and Asthma Control (TRACK) questionnaire, could forecast wheeze attacks over the following three months. Incorporating atopic status or blood neutrophil counts did not enhance the accuracy of the classification performance [25]. Applying these findings in clinical practice may facilitate the identification of at-risk children for whom timely intervention with appropriate treatment can help prevent future episodes.

### 2.4. Patient Engagement and Medical Support

In recent years, various e-Health technology solutions have been introduced to enhance the efficiency of healthcare delivery and promote education and engagement among patients and caregivers.

Mobile applications equipped with AI algorithms are increasingly utilized to deliver therapy reminders, recommendations, and feedback based on data collected from home spirometry or wearable devices, including smart inhalers. These applications can increase adherence to daily treatment, notify parents of potential exacerbations, and propose preventive measures or the need for medical consultation.

For adults and children with asthma, adherence to prescribed treatment should be greater than 75% to be considered acceptable [30]. However, in asthmatic children, estimated treatment adherence rates vary from 49% to 71% [31].

The implementation of smart inhalers in daily practice has the potential to transform asthma management by integrating advanced technology with conventional devices. These inhalers incorporate sensors capable of monitoring and recording both usage patterns and medication adherence, as well as measuring patients’ inspiratory flow. The data collected can be transmitted to a connected application, enabling real-time feedback for both patients and healthcare providers. Furthermore, smart inhalers can deliver reminders to patients regarding medication schedules, thereby reducing the likelihood of missed doses and enhancing overall adherence to asthma treatment regimens. Patient engagement is crucial for the effective management of asthma, as it empowers individuals to take an active role in their own health and treatment decisions. Involved patients are more likely to adhere to medication regimens, recognize and avoid triggers, and monitor their symptoms regularly.

In the Outerspace trial, adult asthma patients who received tailored education and training using a digital smart spacer demonstrated a 26.2% reduction in daily inhalation errors, whereas the usual care group exhibited a 14.6% increase in such errors. Notably, there were no observed changes in lung function between the groups [32].

In children with difficult-to-treat asthma, the use of electronic monitoring with smart inhalers revealed a group of patients who had poor adherence and poor control, suggesting that this subgroup did not have true severe asthma but modifiable factors to address [33]. These findings can be extremely helpful for the clinician to stratify asthma patients and avoid useless increases in asthma therapies.

AI tools are also capable of producing informative content for the public and can act as a supplementary resource in patient education.

Socially, AI-powered robots have already been used in asthma management. They boost motivation and engagement with treatment but also enhance patient education and training [34].

In children robots specifically trained for patients with asthma assist with pre-scribed therapy by offering accurate instructions on what asthma is and act as trainers for inhalation techniques, resulting in general satisfaction among parents and children [35].

These technologies can simplify complex medical information, make personalized recommendations, and support understanding of asthma management strategies.

However, they do not substitute for professional medical advice. It remains essential that healthcare professionals critically evaluate AI-generated information before applying it in clinical practice.

In a recent study, ChatGPT was asked asthma-related questions, and although ChatGPT performed well, the answers were superficial and less accurate than those provided by the specialist [36]. Another study assessed the effectiveness of AI chatbots, including ChatGPT, Bard, and Copilot, in addressing asthma-related inquiries submitted by patients. The findings indicated that while these tools show potential for providing useful information to the public, their accuracy and completeness varied depending on the question, with some tools performing better than others [37].

As digital solutions become increasingly integrated into healthcare practices, their ability to provide reliable help and guidance to clinicians has the potential to improve medical performance, complementing traditional clinical support and resources.

In 167 young children with wheezing, a digital stethoscope was given to the families to recognize wheeze and allow the clinician to make the diagnosis remotely, thereby promoting shared decision-making. These instruments are AI-driven and can identify normal breath sounds, crackles and wheezing not only in the hands of professional operators [38] but also in a home setting [39].

The integration of AI into smart devices such as phones, stethoscopes, or digital inhalers allows physicians to access patient data in real time to provide proactive care, suggesting the right treatment and possibly reducing the risk of loss of control.

AI has also been widely utilized in the analysis of radiological images. ML algorithms are capable of accurately evaluating high-resolution computed tomography (HRCT) scans in adult patients, identifying distinguishing features of severe asthma such as bronchial wall thickness, and stratifying disease severity [40].

In summary, the progressive advancement of AI in asthma care will depend on collaboration among clinicians, researchers, technologists, and patients. As the body of evidence expands and practical experience accumulates, the prudent and responsible incorporation of AI into routine clinical practice may improve pediatric asthma management.

**Figure 1:**
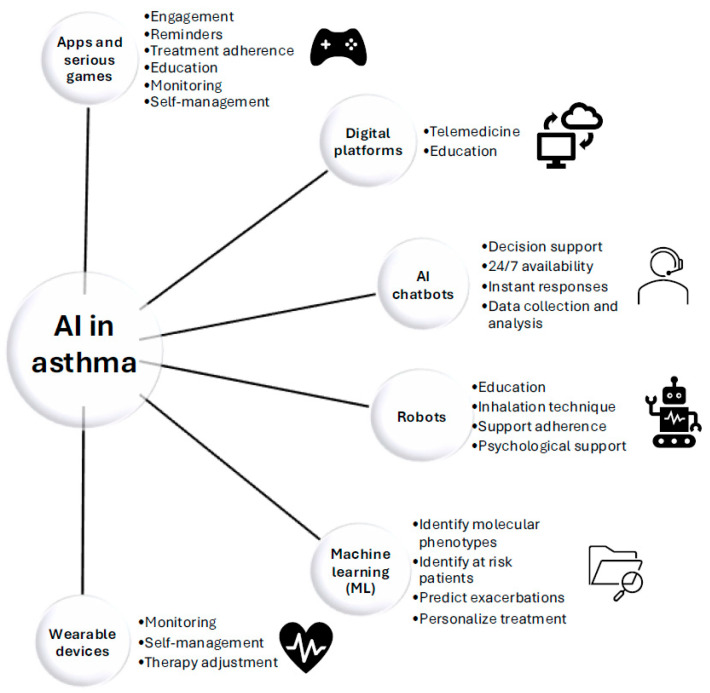
Applications of artificial intelligence (AI) in asthma: examples of apps and serious games, digital platforms, AI chatbots, robots, machine learning, and wearable devices.

Figure 1 illustrates the various applications of AI in the context of asthma.

## 3. Limitations of AI and Barriers to the Implementation of AI in Pediatric Clinical Practice

While AI and ML can enhance asthma diagnosis by analysing large datasets to identify at-risk individuals and precise pheno- and endotypes and can optimize management through wearable devices and patient records, their application in pediatric healthcare presents specific risks and limitations.

ML models, especially those based on deep learning models, can operate as “black boxes,” making it challenging to understand how they arrive at their conclusions. This lack of transparency and interpretability in the decision-making process can lead to incorrect diagnoses, biases, or unfair treatment recommendations. To mitigate these risks, thorough clinical validation is essential. Furthermore, most ML models are trained on adult datasets, which are easier to access and less complex to use and interpret. In contrast, one of the barriers to implementing AI in children with asthma care is the lack of validation of AI in the small and fragmented pediatric datasets. Child-specific datasets are often smaller and less varied than adult datasets; this heterogeneity reduces the accuracy and generalizability of ML models.

Additionally, as children develop, their physiological and developmental characteristics continue to change, which further restricts the generalizability of the data [41]. External validation is essential because models frequently exhibit reduced accuracy when tested on independent datasets, underscoring the need for robust generalizability. Additionally, heterogeneity across datasets, populations, and outcome definitions poses significant challenges, as distribution shifts and inconsistent labeling can lead to biased or unreliable predictions unless mitigated through domain adaptation and fairness-aware strategies [42].

Ethical considerations are also important. ML systems depend on extensive datasets from numerous patients, often sourced through data platforms. This raises important concerns regarding data privacy and security. This is particularly important in vulnerable populations like children and young people due to the complex issue of consent [41].

Some children, particularly minorities or those in underserved areas, often face limited digital literacy and unequal access to technology. Resource limitations in some healthcare systems hinder access to advanced computational infrastructure. In addition, AI applications often require clinicians to interpret algorithmic outputs, integrate predictive insights into decision-making, and adjust workflows accordingly. However, many healthcare professionals have limited exposure to specific training. Training programs are scarce and time-intensive, making it difficult for clinicians to acquire the necessary skills without disrupting clinical duties [43].

The ACCEPT-AI framework seeks to regulate AI use for children and youth by recommending age-appropriate communication, informed consent, data protection, and equity across socioeconomic, racial, and gender factors [44].

## 4. Conclusions

The integration of AI into clinical practice presents both significant opportunities and notable challenges in the management of asthma. The implementation process is complex due to barriers and intrinsic limitations of AI applied to pediatric care. Gaps include limited and fragmented datasets, poor external validation, and a lack of generalizability. Integration into clinical workflows remains challenging due to ethical and regulatory barriers, privacy risks, and insufficient clinician training.

On the other hand, some AI technologies are already offering unique benefits for individuals with asthma. These include apps, web platforms, home spirometers, and smart inhalers that are already deployed in clinical settings and need to be promoted worldwide.

These technologies can improve the accuracy and efficiency of diagnosis, enabling healthcare providers to predict the likelihood of asthma exacerbations, allowing for timely interventions and potentially reducing the severity and frequency of attacks.

Priorities for AI development in pediatric care are integrating AI into the clinical decision system to offer personalized treatment plans. Patients must trust AI technologies and AI-driven tools to enhance engagement and provide support and continuous feedback. Robust validation processes are necessary to confirm the safety and efficacy of AI, supporting informed clinical decision-making.

## Figures and Tables

**Table 1:** Variables taken into consideration by AI algorithms to predict asthma exacerbations.

Demographic Factors	Biomarkers
Ethnicity [22]	Allergy sensitization [22]
Age [22,23,24]	Eosinophils [25]
	Lung function [24,26]
	Respiratory rate [26]
**Clinical history**	**Medications**
Comorbidities (allergic rhinitis, eczema) [22]	Reliever medication usage [26]
Diagnosis of moderate-to-severe asthma [27]	Use of long-acting β agonists [24]
Asthma-related primary care visits [27]	Use of high-dose inhaled steroids [23,24]
Asthma-related emergency department visits [27]	Oral steroid courses [23,24]
Total number of asthma-related visits [22,27]	
Non-asthma-related emergency department visits [27]	**Environmental factors**
Exacerbation in the previous year [28]	Outdoor air quality [27,28]
	Outdoor allergens [28]
	Influenza season [28]

## Data Availability

No new data were created or analyzed in this study. Data sharing is not applicable to this article.
